# Low dose insulin infusion versus the standard dose in children with diabetic ketoacidosis: a meta-analysis

**DOI:** 10.2144/fsoa-2023-0137

**Published:** 2024-05-14

**Authors:** Mohamed Mohamed Belal, Basma Badrawy Khalefa, Eslam Mohammed Rabea, Mazen Negmeldin Aly Yassin, Mohamed Nabih Bashir, Malak Mohamed Abd El-Hameed, Omar Elkoumi, Saad Mohamed Saad, Loubna Mohamed Saad, Mohamed Hamouda Elkasaby

**Affiliations:** 1 Faculty of Medicine, Alexandria University, Alexandria, Egypt; 2 Medical Research Group of Egypt (MRGE), Cairo, Egypt; 3 Faculty of Medicine, Ain shams University, Cairo, Egypt; 4 Faculty of Medicine, Helwan University, Cairo, Egypt; 5 Faculty of Medicine, Zagazig University, Ash Sharqia, Egypt; 6 Faculty of Medicine, Suez University, Suez, Egypt; 7 Faculty of Medicine, Al-Azhar University, Cairo, Egypt

**Keywords:** diabetic ketoacidosis, DKA, insulin, low dose, pediatrics, standard dose

## Abstract

**Aim:** This systematic review aims to consolidate findings from current clinical trials that compare the effectiveness of insulin infusion at 0.05 IU/kg/h versus 0.1 IU/kg/h in managing pediatric diabetic ketoacidosis. **Methods:** We searched several databases, including PubMed, Embase, Scopus, Cochrane Central and Web of Science. Our primary outcomes were time to reach blood glucose ≤250 mg/dl and time to resolution of acidosis. Secondary outcomes included rate of blood glucose decrease per hour, incidence of hypoglycemia, hypokalemia, treatment failure, and cerebral edema. **Results & conclusion:** The present study establishes that a low insulin dose exhibits comparable efficacy to the standard dosage for managing pediatric patients suffering from diabetic ketoacidosis, with a lower incidence of complications.

## Plain language summary

When kids with type 1 Diabetes (T1DM) face a serious complication called Diabetic Ketoacidosis (DKA), it becomes a life-threatening situation. This condition, responsible for significant mortality, involves high blood sugar, ketone buildup and acidity. Our study delves into a critical aspect of DKA treatment-finding the right insulin dose. By pooling the studies on this point, we discovered that using a lower insulin dose is just as effective as the standard dose in managing DKA in children, with fewer complications. This insight is crucial for improving the care and outcomes for young patients dealing with this challenging condition.

## Abstract (plain language summary)

type 1 Diabetes (T1DM) can lead to Diabetic Ketoacidosis (DKA), a severe complication responsible for significant mortality in children.

DKA involves hyperglycemia, ketonemia, and metabolic acidosis and is the initial presentation in about 30% of T1DM children.

Fluid replacement and insulin infusion are core components of DKA management, and the optimal insulin dose has been debated.

High-dose insulin (0.1 IU/kg/h) was once standard, but studies suggest that low-dose (0.05 IU/kg/h) insulin is non-inferior with fewer complications.

The systematic review aimed to compare the effectiveness of low-dose (0.05 IU/kg/h) insulin versus the standard dose (0.1 IU/kg/h) in pediatric DKA.

Five RCTs were analyzed, assessing outcomes like time to reach blood glucose ≤250 mg/dl, acidosis, and incidence of complications.

Low-dose insulin showed comparable efficacy to the standard dose in resolving hyperglycemia and acidosis in pediatric DKA.

Rate of blood glucose decrease was slightly slower with low-dose, but the difference lacked clinical significance.

Low-dose insulin demonstrated a significantly lower incidence of hypoglycemia and hypokalemia compared with the standard dose.

There were no significant differences in treatment failure or the incidence of cerebral edema between the two groups.

The GRADE assessment indicated moderate to high-quality evidence supporting the use of low-dose insulin in pediatric DKA.

Further studies with larger populations and diverse demographics are needed to strengthen the clinical evidence.

More research efforts are warranted to test the efficacy of low-dose insulin in severe cases of DKA separately.

## Graphical abstract

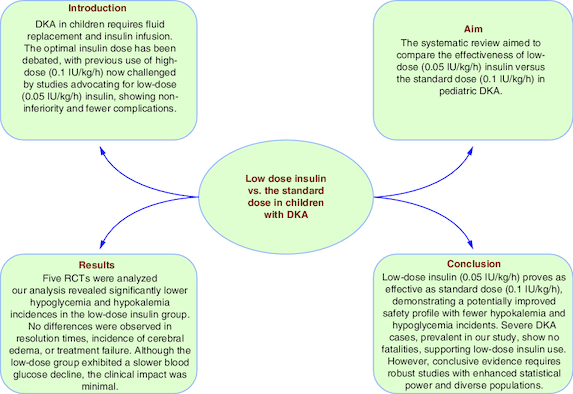

Type 1 diabetes mellitus (T1DM) can lead to a severe and life-threatening complication called diabetic ketoacidosis (DKA). It is the leading cause of death in children with T1DM [1,2]. It is implicated in the mortality of 0.15–0.31% of diabetic children in developed countries [2–4]. However, in developing countries with limited healthcare, mortality ranges from 6 to 24% [5,6]. DKA involves a triad of hyperglycemia (blood glucose >200 mg/dl), ketonemia (amount of ketone bodies in the blood is ≥3 mmol/l), and metabolic acidosis (venous pH <7.3 or serum bicarbonate <15 mmol/l, recently <18 mmol/l) [7,8].

DKA is the initial presentation in about 30% of T1DM children [9]. During a DKA episode, multiple abnormal events occur in the body, including shifting of fluid out of cells, decreased perfusion, and pH changes. Such events affect many bodily functions and lead to electrolyte abnormalities [10,11].

The core components of managing DKA involve replacing fluids and infusing insulin. Initial replacement of fluid loss triggers a substantial decline in hyperglycemia, acidemia and hypertonicity associated with DKA [12]. Gradual correction of fluid loss is important since rapid correction can cause fatal hypokalemia. Moreover, rapid correction of fluid loss can cause significant changes in plasma osmolality rate, increasing the risk of both cerebral and pulmonary edema [13,14]. The addition of intravenous (IV) insulin infusion to initial fluid replacement therapy is essential since it is substantially responsible for peripheral glucose uptake stimulation, suppression of both lipolysis (i.e., partly responsible for acidosis denoted in DKA), and ketogenesis [15,16]. The optimal insulin dose in DKA management has been contested for several decades. Previous high-dose (1 IU/kg/h) and bolus insulin therapies were discontinued after discovering that the same therapeutic effects are possibly attained using a lower dose of 0.1 IU/kg/h in some studies [17,18], which Clinical Practice Consensus Guidelines then established as the standard dose for some time [19].

However, several studies have contested that the administration of low-dose (0.05 IU/kg/h) insulin shows non-inferiority to the standard dose, with the earlier achieving a similar resolution time of ketoacidosis with fewer complications [20–22]: decreased insulin infusion rate gradually reduces glucose levels in the blood, increasing the sodium concentration level in the serum and reducing the effective plasma osmolality [23]; the plasma osmolality change rate is an important factor associated with cerebral edema-the major cause of mortality among pediatrics with DKA [2,24,25].

There is a lack of evidence regarding standardizing the usage of low-dose insulin as a therapy for DKA in the pediatric population. We conducted this review to collect data from available randomized clinical trials (RCTs) regarding the safety, efficacy, and potential implementation of low-dose as standard therapy in treating DKA in the pediatric population.

## Methods

We conducted a systematic review and meta-analysis following the Cochrane guide [26] and the Preferred Reporting Items for Systematic Reviews and Meta-Analyses (PRISMA) statement [27].

### Eligibility criteria

Studies that met the following PICO criteria were included in our meta-analysis: population: patients <18 years with diabetic ketoacidosis; intervention: (0.05 IU/kg/h); comparator: (0.1 IU/kg/h); outcomes: time to resolution of ketoacidosis, and time to resolution of hyperglycemia and study design: randomized controlled trials (RCTs). We excluded non-English studies and observational studies.

### Search strategy

We searched five medical electronic databases, namely PubMed, Embase, Scopus, Cochrane Central, and Web of Science (WOS), from their inception until 2 February 2023.

### Selection of studies

Three authors applied the prespecified inclusion criteria. We used EndNote (version 20 for Windows, PA, USA) [28] to delete duplicates and then used Rayyan [29] website to do title, abstract, and full-text screening. The eligibility screening process involved two steps. The first step involved reviewing titles and abstracts, while the second involved a full-text screening. We also screened the references of included studies for possible missed studies.

### Data extraction

The data was extracted by three authors independently using an online form. If there was a disagreement, it was resolved by census. The extracted data included the following items: study characteristics as 1) study design, duration, and location; 2) characteristics of the study population as age, weight, gender, and biochemical parameters at presentation; 3) risk of bias; and 4) study outcomes.

### Quality assessment

Two authors assessed the RCTs' quality according to the Cochrane Risk of Bias 2 (ROB2) assessment tool [30]. The following items were assessed (randomization process, deviation from intended interventions, missing outcome data, measurement of the outcome and patients, selection of reported bias, and overall bias).

The Grading of Recommendations Assessment, Development and Evaluation (GRADE) scale was used to evaluate the strength and level of evidence for recommendations and was stratified into four categories: high quality, meaning no need for further research and that further research is unlikely to change the confidence of the effects estimations; moderate quality, meaning that more studies can affect the confidence of the effects estimation; low quality, meaning further Research is likely to have a critical impact on the confidence of the effects estimation; and very low quality, meaning that we cannot be sure about those estimations.

### Study outcomes

Our primary outcomes were time to reach blood glucose ≤250 mg/dl in hours and time to resolution of acidosis in hours. Secondary outcomes included the rate of blood glucose decrease, the occurrence of hypoglycemia, hypokalemia, treatment failure, and cerebral edema. The detailed definitions of each outcome in the included studies are presented in Supplementary Table 1.

### Statistical analysis

We utilized Review Manager (RevMan 5.4 for Windows, developed by The Cochrane Collaboration in 2020) [31] and Open MetaAnalyst [32]. RevMan was used for the statistical analysis of time to reach blood glucose ≤250 mg/dl, time to resolution of acidosis, rate of blood glucose decrease, incidence of hypoglycemia, and hypokalemia., while Open MetaAnalyst was used for the statistical analysis of outcomes which included events equal to zero as the incidence of cerebral edema and treatment failure. The results were considered significant if a random error is less than 0.05. The study used the Mantel–Haenszel method to determine the risk ratio and 95% CI for categorical variables described as events and totals. Continuous outcomes were described as (mean and standard deviation), and the mean difference was calculated using the inverse variance method, with a CI of 95%. In the included studies, heterogeneity assessment was done using the I-squared test; there was a significant heterogeneity when the I^2^ was greater than 60% or the p-value was less than 0.1. If there was heterogeneity, we utilized the random effect model. Otherwise, we employed the fixed effect model.

## Results

### Literature search

**Figure 1.**
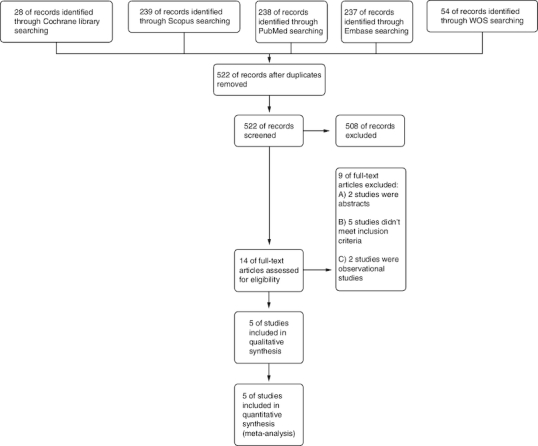
PRISMA flow chart.

We obtained 803 records from various databases as a result of our search. (PubMed: 288, Scopus: 239, Embase: 237, Web of science: 54 and Cochrane: 28). After removing duplicates, 522 remained. Of them, 508 were irrelevant and eliminated during the screening process based on evaluating their titles and abstracts, and 14 records were considered suitable for further evaluation through full-text screening. The meta-analysis incorporated a total of five RCTs, published between 2014 and 2022. No missing studies were discovered after the manual screening of the references of the included studies (see PRISMA flow diagram; Figure 1).

### Characteristics of included studies

**Figure 2.**
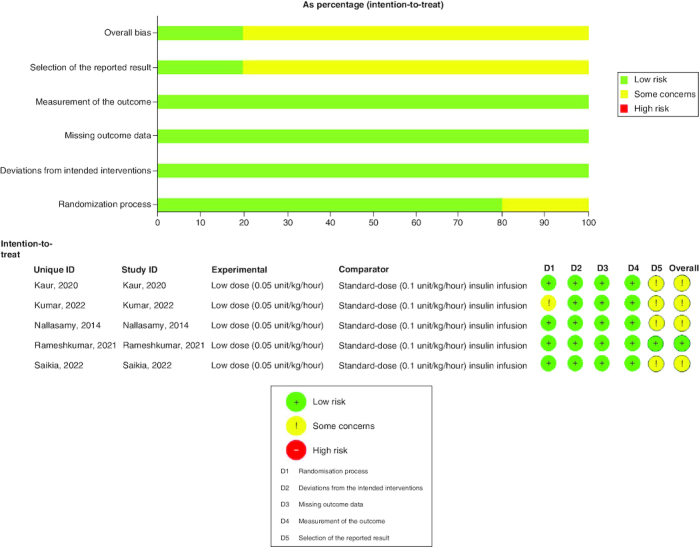
Summary of risk of bias assessment of the included studies.

Five randomized controlled trials conducted in India were included in this study, of which four were open-label and only Nallasamy *et al.* [33] was double-blinded. The study involved 220 patients, with 50% receiving low-dose insulin (0.05 IU/kg/h) and the other 50% receiving the standard dose (0.1 IU/kg/h. Table 1 provides a summary of the key characteristics of the included studies. Patients' baseline data are demonstrated in Table 2. Many of the studies raise concerns regarding the overall risk of bias. We found some concerns in the randomization process of Kumar *et al.* [22] study and the selection of the reported result in four of the included studies. Figure 2 shows the summary of the risk of bias assessment.

**Table 1.** Summary of the included studies.

Study ID (year)	Location	Time	Design	Sample size	Intervention	Comparison	Outcomes	Inclusion criteria	Exclusion criteria
Kaur (2020)	India	June 2018 to September 2019	Open-label randomized controlled trial	50	Low dose (0.05 unit/kg/h)	Standard-dose (0.1 unit/kg/h) insulin infusion	1. Time to reach blood glucose ≤250 mg/dl 2. Time until resolution of acidosis 3. Rate of blood glucose decrease 4. Events of hypoglycaemia 5. Events of hypokalemia. 6. Events of treatment failure 7. Events of cerebral edema	Children 14 years or younger with a diagnosis of DKA	1. Children >14 years. 2. Symptomatic cerebral edema 3. Septic shock at presentation. 4. Anuria for longer than 6 h 5. Received insulin treatment (S.C. or I.V.). before admission
Kumar (2022)	India	March 2017 to August 2018	Open label randomized controlled trial	30	Low dose (0.05 unit/kg/h)	Standard-dose (0.1 unit/kg/h) insulin infusion.	1. Time to reach blood glucose ≤250 mg/dl 2. Time until resolution of acidosis 3. Rate of blood glucose decrease 4. Events of hypoglycemia 5. Events of hypokalemia. 6. Events of treatment failure	Children 12 years or younger with a diagnosis of DKA	Symptomatic cerebral edema
Nallasamy (2014)	India	November 2011 to December 2012.	Open label randomized controlled trial	50	Low dose (0.05 unit/kg/h)	Standard-dose (0.1 unit/kg/h) insulin infusion	1. Time to reach blood glucose ≤250 mg/dl 2. Time until resolution of acidosis 3. Rate of blood glucose decrease 4. Events of hypoglycemia 5. Events of hypokalemia 6. Events of treatment failure 7. Events of cerebral edema	Children 12 years or younger with a diagnosis of DKA	1. Symptomatic cerebral edema 2. Septic shock at presentation 3. Anuria for longer than 6 h 4. Insulin treatment before admission
Rameshkumar (2021)	India	October 2014 to July 2018	Randomized, double-blind controlled clinical trial	60	Low dose (0.05 unit/kg/h)	Standard-dose (0.1 unit/kg/h) insulin infusion	1. Time to reach blood glucose ≤250 mg/dl 2. Time until resolution of acidosis 3. Rate of blood glucose decrease 4. Events of hypoglycemia 5. Events of hypokalemia. 6. Events of cerebral edema	Children 12 years or younger with a diagnosis of DKA	1. Septic shock. 2. Insulin treatment before enrollment
Saikia (2022)	India	–	Open label randomized controlled trial	30	Low dose (0.05 unit/kg/h)	Standard-dose (0.1 unit/kg/h) insulin infusion	1. Time to reach blood glucose ≤250 mg/dl 2. Time until resolution of acidosis 3. Events of hypoglycemia 4. Events of hypokalemia. 5. Events of treatment failure	Children 12 years or younger with a diagnosis of DKA	1. symptomatic cerebral edema 2. septic shock 3. insulin infusion at some other facility before arriving center

DKA: Diabetic ketoacidosis; I.V: Intravenous; S.C: Subcutaneous.

**Table 2.** Baseline characteristics of the enrolled patients in the included studies.

Study ID (year)	Study group	N	Age (years)	Weight (kg)	Gender (males)	New onset DKA, (n)	Established diabetes, (n)	Severe Acidosis	GCS	Initial Blood glucose, mg/dl	Initial PH	Initial bicarbonate, mEq/l	Initial creatinine, mg/dl	Serum sodium, mEq/l	Corrected serum sodium, mEq/l	Effective osmolality, mOsm/kg	Initial Potassium, mEq/l	Anion gap
Kaur (2020)	Standard dose	25	8.78 (4.28)	22.14 (10.55)	11 (44.0%)	10 (40.0%)	15 (60.0%)	19 (76.0%)	–	–	–	–	–	–	–	–	–	–
	Low dose	25	9.72 (3.40)	22.56 (7.84)	10 (40.0%)	10 (40.0%)	15 (60.0%)	18 (72.0%)	–	–	–	–	–	–	–	–	–	–
Kumar (2022)	Standard dose	15	8.30 (2.576)	20.19 (9.07)	4 (26.7%)	10 (66.7%)	5 (33.3%)	–	12.80 (2.541)	–	–	–	–	–	–	–	–	–
	Low dose	15	6.83 (2.678)	17.42 (5.906)	7 (46.7%)	6 (40.0%)	9 (60%)	–	14.27 (1.387)	–	–	–	–	–	–	–	–	–
Nallasamy (2014)	Standard dose	25	6.5 (3.6)	17.3 (7.3)	11 (44%)	16 (64%)	9 (36%)	–	–	524.4 (103)	7.05 (0.11)	7.0 (3.1)	0.7 (0.2)	134.5 (10.0)	141.3 (10.3)	298.2 (21.2)	4.7 (0.7)	28.0 (8.5)
	Low dose	25	7.3 (3.8)	19.2 (9.1)	9 (36%)	13 (52%)	12 (48%)	–	–	485.3 (133)	7.08 (0.12)	6.2 (2.6)	0.7 (0.3)	133.0 (7.0)	138.9 (6.7)	292.0 (13.8)	4.8 (0.8)	27.5 (4.9)
Rameshkumar (2021)	Standard dose	30	8.4 (3.2)	–	–	14 (46.6%)	16 (53.4%)	13 (43.3%)	14.66 (0.74)	510.3 (113)	7.10 (0.16)	7.1 (4.3)	1.1 (0.4)	138 (5.8)	144.6 (5.5)	304.4 (11.3)	3.9 (0.7)	27.5 (5.6)
	Low dose	30	7 (3.6)	–	–	15 (50%)	15 (50%)	10 (33.3%)	14.66 (0.74)	465.5 (105.6)	7.15 (0.13)	8.9 (4.3)	1.0 (0.3)	137.5 (6.2)	143.5 (6.8)	292.4 (45.8)	3.9 (0.7)	26 (7.4)
Saikia (2022)	Standard dose	15	8.30 (2.576)	20.19 (9.07)	4 (26.67%)	12 (80%)	3 (20%)	11 (73.3%)	12.8	478.5 (49.5)	6.96 (0.338)	7.28 (3.580)	0.63 (0.23)	–	–	–	–	–
	Low dose	15	6.83 (2.678)	17.42 (5.906)	7 (46.67%)	12 (80%)	3 (20%)	10 (66.67%)	14.2	491.7 (49.7)	7.04 (0.141)	6.36 (3.475)	0.67 (0.22)	–	–	–	–	–

GCS: Glasgow Coma Scale.

### Outcomes

#### Time to reach blood glucose ≤250 mg/dl

**Figure 3.**
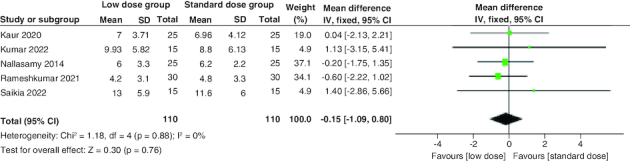
A Forest plot of the time to reach blood glucose ≤250 mg/dl.

The combined mean difference (MD) from the included studies indicated a non-significant distinction between the two groups concerning the duration required for the resolution of hyperglycemia (MD = -0.15 hr; 95% CI: [-1.09–0.8], p = 0.76). The pooled studies were homogenous (p = 0.88; I^2^ = 0%) Figure 3.

#### Time to resolution of acidosis

**Figure 4.**
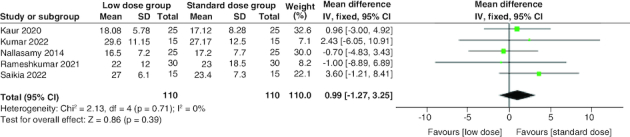
Forest plot of the time to resolution of acidosis (h).

The pooled analysis of the five studies revealed that the standard dose group had a shorter time to resolve acidosis than the low dose group. However, the difference was statistically insignificant (MD = 0.99 hr; 95% CI: [-1.27–3.25], p = 0.39). There was no significant heterogeneity among studies (p = 0.71; I^2^ = 0%) Figure 4.

#### Rate of blood glucose decrease

**Figure 5.**
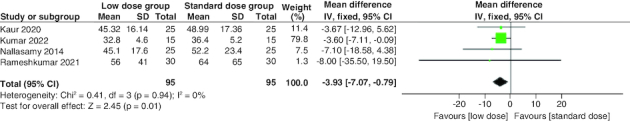
Forest plot of rate of blood glucose decrease until level is ≤250 mg per dl per hour.

As shown in Figure 5, the low-dose group showed a lower statistically significant rate of blood glucose decrease compared with the standard dose group (MD = -3.93 mg/dl/h.; 95% CI: [-7.07–-0.79], p = 0.01), but the difference was clinically not significant. The combined studies were homogenous (p = 0.94; I^2^ = 0%).

#### Incidence of hypokalemia

**Figure 6.**
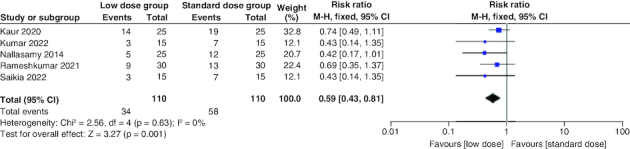
Forest plot of the incidence of hypokalemia.

The pooled risk ratio (RR) of the included studies favored the low-dose group, which had a significantly lower risk of hypokalemia than the standard-dose arm (RR = 0.59; 95% CI: [0.43–0.81], p = 0.001). We observed no significant heterogeneity among studies (p = 0.63; I^2^ = 0%) Figure 6.

#### Incidence of hypoglycemia

**Figure 7.**
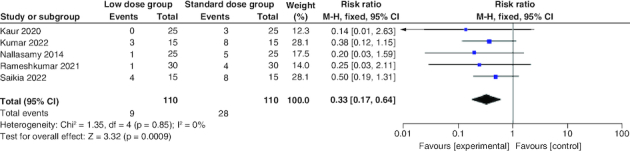
Forest plot of the incidence of hypoglycemia.

Regarding the incidence of hypoglycemia, the combined RR revealed a statistically significant lower rate in the low-dose arm compared with the standard-dose arm (RR: 0.33; 95% CI: [0.17–0.64], p = 0.0009). The pooled studies were homogenous (p = 0.85; I^2^ = 0%) Figure 7.

#### Treatment failure

**Figure 8.**
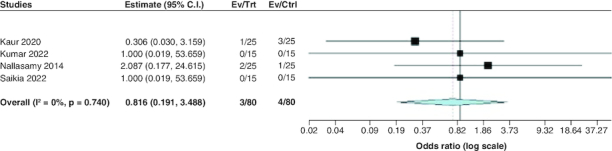
Forest plot of the incidence of treatment failure.

The pooled OR of the included studies revealed a statistically insignificant difference between the two groups regarding treatment failure (OR: 0.82; 95% CI: [0.19–3.49], p = 0.78). We observed no significant heterogeneity among studies (p = 0.74; I^2^ = 0%) Figure 8.

#### Incidence of cerebral edema

**Figure 9.**
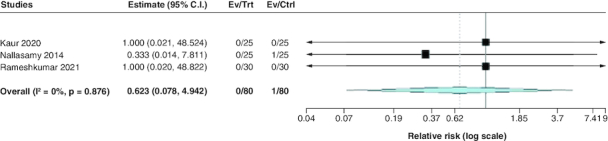
Forest plot of the incidence of cerebral edema.

Regarding the incidence of cerebral edema, the pooled estimate of the included studies showed a statistically insignificant difference between both groups (RR: 0.62; 95% CI: [0.08–4.94], p = 0.65). We observed no significant heterogeneity among studies (p = 0.88; I^2^ = 0%) Figure 9.

### GRADE assessment

The GRADE rating results are shown in Supplementary Table 2. According to the GRADE system, the strength of evidence was moderate for all reported outcomes except for hypoglycemia for which the strength of evidence was high.

## Discussion

In our study, which included five RCTs with 220 participants, we found that the incidences of hypoglycemia and hypokalemia were significantly lower in the low-dose group than in the standard-dose group. There were no differences in time to resolution of ketoacidosis, time to reach blood glucose ≤250 mg/dl, incidence of cerebral edema, and treatment failure. The rate of blood glucose decline per hour was significantly slower in the low-dose group, but the difference was too minimal to have any clinical impact (MD = -3.93 mg/dl/h).

Cerebral edema, hypoglycemia and hypokalemia may cause death in children treated for diabetic ketoacidosis, so the incidence of those complications is of clinical importance. Insulin causes shifting of glucose from plasma to the intracellular compartment leading to a drop in plasma osmolarity. A rapid drop in plasma osmolarity may be the driving factor contributing to cerebral edema incidence [34]. Recent guidelines have moved on using insulin infusion during the first hour of therapy because fluids alone were found to cause a blood glucose decrease of around 109 mg/dl [35].

In theory, low-dose insulin (0.05 IU/kg/h) may lead to a more gradual decrease in blood glucose and a more gradual decline in plasma osmolarity. This may lead to less incidence of cerebral edema. However, there was no significant difference between the two groups. No difference in incidence of cerebral edema can be attributed to the limited number of included patients. Also, most of the included studies have excluded patients with baseline cerebral edema [21,22,33,36].

The incidence of hypokalemia was significantly lower in the low-dose group. However, none of the studies included in the analysis reported a significant difference between both groups regarding the incidence of hypokalemia. The difference between the aggregated analysis findings and those of individual studies underscores the significance of this meta-analysis.

The frequency of hypoglycemic episodes was markedly reduced in the low-dose group. Our results align with the findings reported by Puttha *et al.* [37]. While individual studies did not show a significant difference in the incidence of hypoglycemia, our meta-analysis has demonstrated the statistical significance of aggregated data. Malnutrition is an aggravating factor for hypoglycemia. Moulik *et al.* found that the incidence rate of hypoglycemia was 30.3%; however, this rate increased to 64% when the malnourished children were considered alone [38], so the difference in results can be due to the baseline nutritional status. Insulin causes hypoglycemia by causing intracellular shifting of glucose into muscle and adipose tissue [39]. The confounding attributed to difference in nutritional status between both groups was reduced as both the low and the standard dose groups have the same baseline characteristics regarding nutritional status. In Rameshkumar *et al.*, a total of 16 out of 60 children had baseline malnutrition (7 in the low dose group vs 9 in the standard dose group) [20], Fifteen out of 50 children in Nallasamy *et al.*, (7 in the low dose vs 8 in the standard dose group) [33] and 12 out of 30 in Kumar *et al.*, (6 in the low dose vs 6 in the standard dose group) [22]. Also, difference in fluid protocol used could be a contributing factor in hypoglycemia, but the included studies had no major differences in fluid protocols used. Further information regarding fluid resuscitation provided in Supplementary Table 1.

The time to resolution of acidosis and time to reach blood glucose ≤250 mg/dl were also similar between both groups. Those findings are consistent with what was found in a cohort study conducted by Puttha *et al.* [37].

The decrease in blood glucose levels during insulin therapy can be attributed to three processes: suppression of hepatic glucose production, increased tissue intake of glucose, and renal excretion of glucose, with suppression of hepatic glucose production being the most important process [16]. In patients with DKA, hepatic tissue exhibits some resistance to insulin action. So, higher insulin doses are required to overcome this resistance (80–100 μU/ml) [40]. It was reported in some studies that the (0.1 IU/kg) dose achieved plasma level (100–200 IU/ml) [18,41]. This may be why hyperglycemia can be resolved by doses lower than (0.1 IU/kg). The plasma insulin level wasn't measured in the included studies, but the results suggest that even low-dose insulin can reach physiological levels sufficient to suppress hepatic glycogenolysis and gluconeogenesis.

The rate of blood glucose decline per hour was significantly lower in the low-dose group; however, this does not reach clinical significance (MD = -3.93 mg/dl/h). Treatment failure wasn't significantly different between both groups. We examined the included studies to identify data on mortality, but such information was unavailable.

A cohort study performed by Al Hanshi *et al.*, found that both low and standard dose groups have similar length of stay in the intensive care unit [42]. Length of hospital stay (LOS) wasn't mentioned in any of the included studies. It would be beneficial to include it as a secondary outcome in future studies as it will reflect the incidence of complications and time needed to treat those complications.

This systematic review and meta-analysis pooled the available evidence regarding the use of low-dose insulin in cases of pediatric DKA instead of the standard dose. The studies included being homogenous to a great extent in terms of populations, interventions, controls, and outcomes, reflecting statistically homogenous results. Our results go with the latest guidelines of the International Society of Pediatric and Adolescent Diabetes, which recommended a correction dose of insulin to be in a range of 0.05–0.1 U/kg/h with the consideration of the lower dosage (0.05 U/kg/h) only when pH >7.15 (8). More research efforts are warranted to test the efficacy of low-dose insulin in severe cases of DKA.

## Limitations

The number of included studies and participants of each study was greatly limited. In addition, all included studies were found to originate from one country (India), limiting the concluded results' certainty and generalizability. Four of the five included studies were designed as open-label trials, which might represent a source of potential bias. Such a case has contributed to the overall quality of the included studies having some concerns. One of the limitations in our study is the lack of data concerning the classification of children into categories of mild, moderate, and severe DKA. Additionally, Baseline PH and glucose level wasn't reported in 2 of the included studies. Another drawback in our analysis is that imprecision was serious in all included studies due to paucity of included patients and low number of events.

## Conclusion

Using the low dose insulin (0.05 IU/kg/h) is as effective as the standard dose insulin (0.1 IU/kg/h) in the pediatric population with diabetic ketoacidosis, perhaps with a better safety profile regarding the incidences of both hypokalemia and hypoglycemia. Children with severe DKA constitute a considerable proportion of patients in the included studies with no fatalities reported. This encourages further exploration of low-dose insulin, even in severe cases. However, to establish conclusive clinical evidence, additional studies with increased statistical power and diverse populations are essential.

## Supplementary Material

Supplementary Tables S1-S2
