# Supplementary material for: Low dose insulin infusion versus the standard dose in children with diabetic ketoacidosis: a meta-analysis
Source: Future Sci OA. 2024 May 14;10(1):FSO956. doi: 10.2144/fsoa-2023-0137 (PMC11140676; doi:10.2144/fsoa-2023-0137)
Supplement: Supplementary Tables S1-S2 [file IFSO_A_2340192_SM0001.docx]

**Table (S1), Outcomes definitions and Fluid protocol.**

| **Study ID** | **Hypoglycemia** | **Hypokalemia** | **Treatment failure** | **Severe acidosis** | **Fluid protocol used** |
| --- | --- | --- | --- | --- | --- |
| **Kaur 2020** | Blood glucose level less than 54 mg/dL | Serum potassium level less than 3.5 mEq/L | NR | NR | NR |
| **Saikia 2022** | Blood glucose level of 70 mg/dL or less | Serum potassium less than 3.5 mEq/L | Failure to achieve a reduction in blood glucose of 18 mg/dl per hour for 2 consecutive hours and/or a decrease or failure of rise in serum bicarbonate with persistent high anion gap acidosis | pH < 7.1 and serum bicarbonate < 5 mEq/L | The fluids were calculated and administered as per recommendations, beginning with an initial fluid bolus, followed by uniform infusion at the calculated rate. |
| **Nallasamy 2014** | Blood glucose level of 60 mg/dL or less | Serum potassium level less than 3.5 mEq/L and/or suggestive electrocardiographic changes. | Failure to achieve a blood glucose reduction of 18 mg/dL per hour for 2 consecutive hours and/or a decrease or failure to increase in bicarbonate with persistent high anion gap acidosis | pH < 7.1 or bicarbonate < 5mEq/L | -Fluid volume was calculated as a sum of deficit (65 mL/kg) and maintenance for 36 hours.  -Children who presented with evidence of hypoperfusion or hypotensive shock received an additional 20mL/kg of isotonic saline for 1 hour.  - Isotonic (0.9%) saline was used as rehydrating fluid for at least the initial 6 hours and changed to half normal (0.45%) saline based on serum sodium and corresponding effective osmolality.  -Dextrose (5%) was added to rehydrating fluid once the BG level decreased to 250 mg/dL or less and the concentration titrated to maintain a BG level between 180 and 220 mg/dL.  - Potassium chloride (40 mEq/L) was added to rehydrating fluid immediately after resuscitation to maintain a serum potassium level between 3.5 and 5.5 mEq/L. |
| **Rameshkumar 2021** | Blood glucose level of 60 mg/dL or less | Serum potassium less than 3.5 mEq/L and/or suggestive electrocardiographic changes | NR | NR | -Fluid volume was calculated as a sum of the deficit (85 mL/kg) and 48-hour maintenance fluid spread over 48 hours.  - All children received 20 mL/kg of normal saline in the first hour of resuscitation. Children with evidence of hypoperfusion or hypotensive shock received an additional 20 mL/kg of normal saline for one hour. The bolus and other infusions were deducted from the total calculated volume to be infused.  - Normal saline was used for the first six hours and was changed to 0.45% saline based on serum sodium and effective osmolality.  - Dextrose (5%) was added to the hydrating fluid once the blood glucose level decreased to 250 mg/dL or less. If the blood glucose level approached 100 mg/dL or below despite dextrose concentration of 12.5%, the insulin infusion was tapered at the rate of 0.01 mL/kg/hour every half-hourly.  - Potassium chloride (40 mEq/L) was added to the rehydrating fluid after resuscitation and documentation of urine output.  - Continuous cardiac monitoring was done and potassium titrated to maintain a serum level of 3.5-5.5 mEq/L. |
| **Kumar 2022** | NR | NR | NR | NR | NR |

**Abbreviations:** DKA, diabetic ketoacidosis. I.V, Intravenous.

**Table (S2), Grade assessment.**

|  | | | **Certainty assessment** | |  |  | **№ of patients** | | **Effect** | | **Certainty** | **Importance** |
| --- | --- | --- | --- | --- | --- | --- | --- | --- | --- | --- | --- | --- |
| **№ of studies** | **Study design** | **Risk of bias** | **Inconsistency** | **Indirectness** | **Imprecision** | **Other considerations** | **. 05 IU/kg** | **. 1 IU/kg** | **Relative (95% CI)** | **Absolute (95% CI)** |  |  |
| **Time of resolution of Acidosis** | | |  | |  |  |  | |  | |  |  |
| 5 | randomised trials | not serious | not serious | not serious | Serious ^1^ | none | 110 | 110 | - | MD **0.99 higher**  (1.27 lower to  3.25 higher) | ⨁⨁⨁◯  Moderate | CRITICAL |

**Time of resolution of hyperglycemia**

| 5 | randomised trials | not serious | not serious | not serious | Serious^1^ | none | 110 | 110 | - | **0.15 lower**  (1.09 lower to  0.8 higher) | ⨁⨁⨁◯  Moderate | IMPORTANT |
| --- | --- | --- | --- | --- | --- | --- | --- | --- | --- | --- | --- | --- |

**Hypokalemia**

| 5 | randomised trials | not serious | not serious | not serious | Serious^1^ | none | 34/110 (30.9%) | 58/110 (52.7%) | **RR 0.59**  (0.43 to 0.81) | **216 fewer per**  **1,000**  (From 301 fewer to 100 fewer) | ⨁⨁⨁◯  Moderate | IMPORTANT |
| --- | --- | --- | --- | --- | --- | --- | --- | --- | --- | --- | --- | --- |

**Hypoglycemia**

| 5 | randomised trials | not serious | not serious | not serious | Serious^1^ | strong association | 9/110 (8.2%) | 28/110 (25.5%) | **RR 0.33**  (0.17 to 0.64) | **171 fewer per**  **1,000**  (From 211 fewer to 92 fewer) | ⨁⨁⨁⨁  High | IMPORTANT |
| --- | --- | --- | --- | --- | --- | --- | --- | --- | --- | --- | --- | --- |

**Treatment failure**

| 4 | randomised trials | not serious | not serious | not serious | Serious^2^ | none | 3/80 (3.8%) | 4/80 (5.0%) | **OR 0.816** (0.191 to 3.488) | **9 fewer per**  **1,000**  (From 40 fewer to 105 more) | ⨁⨁⨁◯  Moderate | IMPORTANT |
| --- | --- | --- | --- | --- | --- | --- | --- | --- | --- | --- | --- | --- |

**Cerebral edema**

| 3 | randomised trials | not serious | not serious | not serious | Serious^2^ | none | 0/80 (0.0%) | 1/80 (1.3%) | **OR 0.623** (0.078 to 4.942) | **5 fewer per**  **1,000**  (From 12 fewer to 46 more) | ⨁⨁⨁◯  Moderate | CRITICAL |
| --- | --- | --- | --- | --- | --- | --- | --- | --- | --- | --- | --- | --- |

**Rate of blood glucose decrease per hour till equal or less than 250 mg/dl.**

| 4 | randomised trials | not serious | not serious | not serious | serious | none | 95 | 95 | - | MD **3.93 lower**  (7.07 lower to  0.79 lower) | ⨁⨁⨁◯  Moderate | NOT IMPORTANT |
| --- | --- | --- | --- | --- | --- | --- | --- | --- | --- | --- | --- | --- |

**CI:** confidence interval; **MD:** mean difference; **OR:** odds ratio; **RR:** risk ratio

^1^Due to the relative few number of the included patients.

^2^Due to the few number of included patients and insufficient events across the included studies
